# Investigation of Antibody Pharmacokinetics in Male Reproductive System and Its Characterization Using a Translational PBPK Model

**DOI:** 10.3390/antib14010017

**Published:** 2025-02-13

**Authors:** Sree Ojili, Dhaval K. Shah

**Affiliations:** Department of Pharmaceutical Sciences, School of Pharmacy and Pharmaceutical Sciences, The State University of New York at Buffalo, Buffalo, NY 14214-8033, USA

**Keywords:** antibody, pharmacokinetics, male reproductive system, PBPK modelling, clinical translation

## Abstract

**Objectives:** To investigate the pharmacokinetics (PK) of the monoclonal antibody (mAb) in male reproductive tissues and develop a translational physiologically based pharmacokinetic (PBPK) model to characterize the PK data. **Method:** The PK of a non-cross-reactive antibody (trastuzumab) was investigated in human FcRn-expressing male mice following a 10 mg/kg intravenous dose. The PK in plasma and male reproductive tissues (i.e., epididymis, testes, vas deferens, seminal vesicles, and prostate glands) were evaluated. The observed PK data in mice were mathematically characterized using a novel PBPK model for antibodies that contained male reproductive systems. The mouse PBPK model was scaled to rats, monkeys, and humans to predict the PK of antibodies in male reproductive organs across animal species. **Results**: Plasma and tissue PK data generated in mice suggest that antibody distribution in male reproductive tissues is generally lower compared to that of most of the organs. The antibody exposure in the testes was 1.70%, in the epididymis was 2.57%, in the vas deferens was 2.01%, in the seminal vesicle was 0.42%, and in the prostate gland was 0.52% of the plasma exposure. The plasma and tissue PK data were simultaneously characterized using the PBPK model, which incorporated the novel male reproductive system. All the predicted PK profiles were within two-fold of the observed data, as indicated by percentage prediction error (%PE) values. The mouse model was successfully translated to bigger animals, and the model was used to simulate the PK of antibodies in rat, monkey, and human male reproductive systems. **Conclusions**: The combination of the experimental data and novel PBPK model presented here provides unprecedented insights into the antibody distributions in different male reproductive tissues. The PBPK model can serve as a crucial tool for advancing the development of antibody-based therapies for treating sexually transmitted infections (STIs), cancers, and contraceptives.

## 1. Introduction

Globally, approximately 400 million new cases of sexually transmitted infections (STIs) are reported annually, contributing significantly to morbidity and mortality. Although the wide array of antiretroviral drugs has demonstrated remarkable efficacy in controlling plasma viremia, their ability to fully eliminate the virus from certain tissues remains limited. Specifically, viral load within the testes often remains elevated compared to levels in the serum or plasma, even after treatment [1]. Antiviral therapy effectiveness largely depends on the drug’s ability to penetrate tissue-specific barriers, such as the blood–testis barrier, and neutralize the viruses hidden within these protected sites. This challenge has shifted the focus towards the use of monoclonal antibodies (mAbs) that possess the ability to cross these barriers by binding to the FcRn receptors located in the mucosal membranes of various organs. These antibodies offer a promising therapeutic strategy not only for the HIV and Zika virus but also for different types of reproductive-related cancers. Additionally, mAbs hold potential as immunocontraceptives, offering a novel approach to reproductive health by targeting the reproductive system and preventing conception.

To date, the FDA and EMA have approved at least 137 mAbs for clinical use. The World Health Organization (WHO) has acknowledged the critical role of mAbs in preventing HIV and other sexually transmitted infections (STIs). Recently, numerous broadly neutralizing antibodies (bnAbs) have been investigated for their potential use in both the prevention and management of HIV [2]. Of the 137 approved mAbs, approximately 50 have demonstrated antiviral activity, with three advancing to clinical trials.

Before advancing the research and development of mAbs for the prevention and treatment of sexually transmitted infections (STIs), it is essential to thoroughly understand the pharmacokinetics (PK) and tissue distribution of these antibodies within the male reproductive system, including organs such as the testes, epididymis, vas deferens, seminal vesicles, and prostate gland. Understanding how mAbs are distributed within these tissues is critical for optimizing their therapeutic efficacy and ensuring they can effectively target and neutralize pathogens in these anatomically complex sites.

A key approach to better understand the tissue distribution of antibodies is the development of mathematical models, such as physiologically based pharmacokinetic (PBPK) models, to characterize the PK data. PBPK models integrate the physicochemical properties of a drug with the physiological characteristics of the biological system to predict drug disposition. PBPK models, which are widely utilized in drug development, provide a mechanistic framework that allows for the simulation of drug behavior in specific tissues. Their application has steadily increased and is now widely accepted by regulatory bodies such as the FDA [3]. Moreover, PBPK models are valuable tools in the planning and refinement of reproductive and developmental toxicology studies, making them indispensable for understanding mAb disposition in the reproductive system and guiding the development of more effective prevention and treatment strategies [4].

To better understand the distribution of antibodies in the male reproductive system, we first conducted biodistribution studies using a non-binding antibody in human FcRn-expressing male mice. The PK of antibodies in plasma and key organs such as the testes, epididymides, vas deferens, seminal vesicles, and prostate gland were evaluated. The mouse biodistribution data were characterized using a PBPK model that included a novel male reproductive system compartment for antibody disposition. The developed mouse PBPK model was scaled to rats, monkeys, and humans to facilitate a comprehensive understanding of antibody disposition in male reproductive system. While a prior study developed a PBPK model to describe drug distribution in the testes, it did not account for large-molecular-weight compounds, such as mAbs, within the testes or other regions of the male reproductive system [5]. Furthermore, this model did not adequately capture the complexities of mAb distribution across the various compartments of the reproductive system. Thus, this manuscript presents unprecedented PK data and a PBPK model to characterize antibody disposition in the male reproductive system.

## 2. Materials and Methods

### 2.1. Antibody Production and Characterization

Trastuzumab, an anti-human HER2 antibody with no cross-reactivity to mice, was used for the mouse biodistribution experiments. The antibody was produced in-house using CHO cells, whose origin has been published before [6,7]. CHO cells stored in liquid nitrogen were thawed and their viability was measured to generate antibodies. The cells were resuspended in 5 mL of CD–CHO medium and centrifuged at 100× *g* for 5 min if the viability was ≥90%. The pellet was resuspended in new CD–CHO media with 1% Pen-Strep after the supernatant was disposed of. Bacterial contamination and cell viability were checked every other day. The cells were scaled up to a final volume of 800 mL upon reaching the appropriate confluency. Purification of the antibodies started after half of the cells had died. After removing debris with a centrifuge set at 10,000 rpm for 20 min at 4 °C, the culture media was filtered through 0.45-µm and 0.22-µm filters. Antibodies were purified using Protein G column chromatography, with the column being equilibrated in phosphate buffer (pH 7.0) and washed with glycine buffer (pH 2.7) for elution. Eluted antibodies were neutralized with Tris–HCl, and the buffer was exchanged three times with 1Xdpbs (Gibco™ 14200075, Catalogue No: 14-200-075). The concentration was measured at OD280 using Nanodrop (Thermo Fisher Scientific Inc., Waltham, MA, USA), and the antibodies were stored at 4 °C. To check the purity of the produced antibody, SDS–PAGE was performed under reducing and non-reducing conditions. For the reducing sample, a mixture of reducing buffer, diluted antibody, 2-mercaptoethanol, and water was first heated at 95 °C for 10 min and then cooled. The non-reducing sample was prepared by adding a non-reducing buffer and making the final volume 10 μL. Both samples were briefly centrifuged and loaded into an SDS–PAGE gel, which was run at 100 V for about an hour. Afterward, the gel was stained with Coomassie Blue(G250), destained overnight, and read using a Bio-Rad system (Bio-Rad, Hercules, CA, USA) to check the purity of the produced antibody.

### 2.2. PK Study to Investigate the Distribution of Antibodies in the Mouse Male Reproductive System

Every animal experiment was authorized by the Institution of Animal Welfare and Use Committee (IACUC) and carried out in complete accordance with laboratory animal care regulations. Twelve male hFcRn-expressing Tg32 homozygous mice (The Jackson Laboratory, Bar Harbor, ME, USA) were used for the biodistribution study. Four groups of three mice each were created, each of which represented a distinct time point. Mice were given a single intravenous injection of trastuzumab at a dose of 10 mg/kg. At predetermined time points (6, 24, 96, and 168 h), three animals per group were sacrificed following perfusion. Plasma and reproductive organs, including epididymis, testes, vas deferens, seminal vesicles, and prostate glands, were harvested. Antibody concentrations in both plasma and tissues were quantified using sandwich ELISA.

### 2.3. Analytical Method Development

A sandwich ELISA was employed to quantify antibody drug concentrations in plasma and reproductive organs using 384-well plates (Thermoscientific, Catalogue No:464718) with a maximum working volume of 100 µL per well. Each well was coated with 60 µL of 5 µg/mL Goat anti-human IgG (Fc-specific, cross-absorbed) in 20 mM NaHPO₄ (PH-7.5) (and centrifuged at 1000 rpm for 1 min, followed by overnight incubation at 4 °C. On the day of the assay, plates were brought to room temperature, washed three times with 1X PBS + 0.05% TWEEN 20, and washed three additional times with distilled water. Blocking was performed by adding 90 µL of blocking buffer per well, followed by a 2-h incubation. Standards and quality controls were prepared by diluting control plasma 1000-fold, while plasma samples were diluted 10,000-fold. Tissue samples (testis, seminal vesicles, epididymis, prostate) were homogenized in RIPA buffer with protease and phosphatase inhibitors (Thermo Scientific™78441, catalogue Number-PI78441) using a bead homogenizer, while smaller tissues (vas deferens) were sonicated, achieving an 80-fold dilution. To mitigate matrix effects, standards, quality controls, and samples were prepared from tissue homogenates and incubated overnight at 4 °C. On the day of analysis, standards and controls were further diluted in 1X PBS and centrifuged at 13,000 rpm for 15 min. Then, the supernatant was collected. After washing the plates, 30 µL of standards, quality controls, and samples were added to the wells, centrifuged at 1000 rpm for 1 min, and incubated for 2 h. Plates were rewashed, and 30 µL of the detection antibody (1:2500 in wash buffer) was added and incubated for 1 h. After a final wash, 60 µL of p-nitrophenyl phosphate (1 mg/mL in diethanolamine buffer) was added to each well, and absorbance at 405 nm was measured using a Filtermax^TM^ FM microplate reader. The data were interpreted using a 5-parameter standard curve with SoftMax Pro software (v6). Optimized standard curves for each tissue are available in the Supplementary Material (Figure S1).

### 2.4. Data Analysis

To get a clear understanding of the exposure of the antibody in each male reproductive tissue, antibody biodistribution coefficient (ABC) values were calculated using the area under the concentration–time curve (AUC) for both tissues and plasma. AUC values were determined from time 0 to the last observed time point using PKanalix 2023R1. The ABC values for each tissue were derived by taking the ratio of the tissue AUC to the plasma AUC, as shown in the equation below [8].

$$ABC=\frac{AUC_{tissue}}{AUC_{plasma}}\times 10$$

### 2.5. PBPK Model Structure

**Figure 1:**
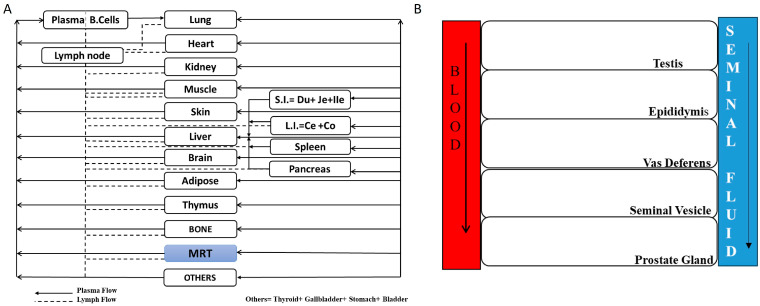
(**A**)**: Schematic of the whole-body PBPK model**: This figure provides a schematic view of the whole-body PBPK model, illustrating how organs are anatomically connected through plasma and lymphatic flow. Each organ is depicted as a rectangular compartment, with solid lines representing plasma flow and dotted lines indicating lymph flow. Organs not explicitly included in the PBPK model are grouped into the “other” compartment, ensuring a comprehensive representation of the body’s physiology. (**B**)**: Schematic representation of the male reproductive system**: This figure illustrates the male reproductive system, highlighting key structures such as the testes, epididymis, vas deferens, seminal vesicles, and prostate gland. The schematic traces the movement of luminal fluid from sperm production within the seminiferous tubules through the reproductive ducts and, ultimately, to ejaculation. The anatomical connections between these organs, as well as the fluid dynamics involved, are represented, providing a detailed overview of the system’s functional flow.

A previously established platform PBPK model for antibodies published by Shah and Betts [9] was expanded to include the male reproductive tissues (MRTs). The original model consisted of 15 organs, as shown in Figure 1A (heart, lungs, adipose tissue, thymus, pancreas, small intestine, large intestine, liver, bone, skin, and brain, and all the remaining organs were grouped into the ‘other’ compartment). Each organ mentioned above was divided into four compartments: vascular, endothelial, interstitial, and cellular. Vascular compartment was further divided into blood cells and plasma sub-compartments. When mAb molecules entered the vascular space, they were taken up by endothelial cells through pinocytosis (CLup) or transported to the interstitial space with the help of the lymphatic flow and reflection coefficient, which represented the resistance offered by the vascular membrane. Once the antibody molecule reached the endosomal space of the endothelium, it could bind to the neonatal Fc receptor (FcRn) using the association rate constant (kon_FcRn) and dissociation rate constant (koff_FcRn). Unbound antibody molecules could be degraded, which was represented by the degradation rate constant (kdeg), while those bound to FcRn could either be recycled back to the plasma (recycling fraction, FR) or sent to the interstitial space (1—FR). From the interstitial space, the antibody molecules could be taken up again by the endothelial cells, or they could exit the compartment by lymphatic flow, where the interstitial reflection coefficient represented the level of resistance for the movement of the mAb molecules into the lymphatic system. If antigens were present in the cellular compartment, they could bind to antibody molecules and influence their behavior in the body.

**Figure 2:**
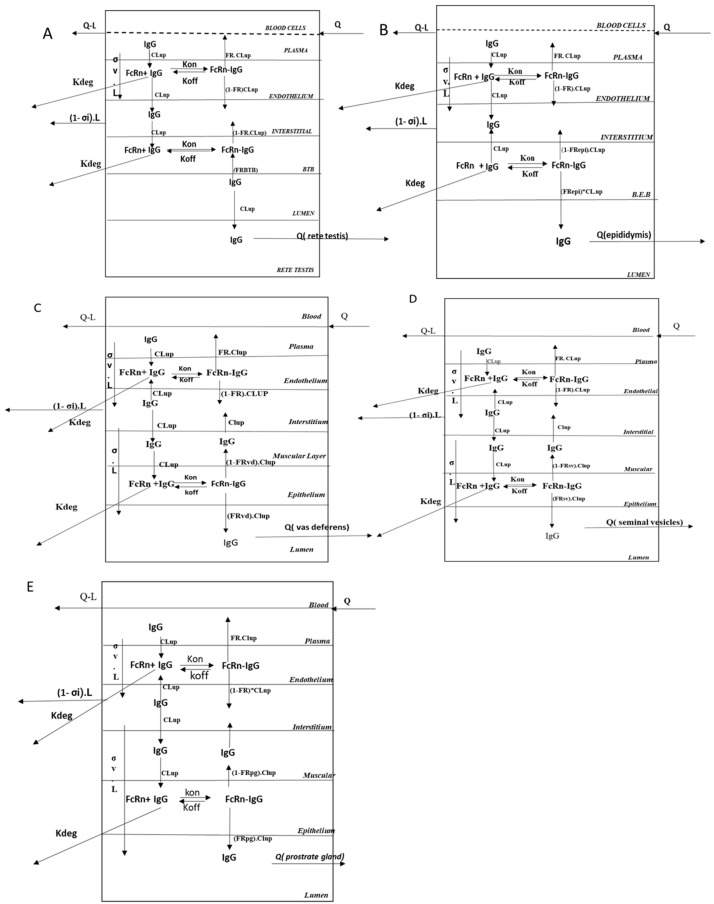
Tissue-level representation of the male reproductive system. All the tissues are connected with the blood, lymph flow, and seminal fluid flowing through the lumen of each tissue. Each tissue is further divided into 4 to 5 sub-compartments based on the anatomy of the tissue. (**A**) Testis, (**B**) Epididymis (EP), (**C**) Vas Deferens (VD), (**D**) seminal vesicle (SV), and (**E**) Prostate Gland (PG).

To characterize the distribution of mAbs in the male reproductive system, we first developed tissue-specific PBPK models for key organs, such as the testes, epididymis, vas deferens, seminal vesicle, and prostate gland (Figure 1B and Figure 2). These models were later integrated into the whole-body PBPK model described above. Each organ was subdivided into five compartments, namely, vascular, interstitial, epithelial (or barrier), and luminal compartments, based on its anatomical structure. Drug distribution within these organs followed the same pattern as described above for each organ. However, after entering the interstitial space, the mAb was allowed to diffuse into the epithelial barrier, where it bonded to FcRn receptors. Following receptor binding, the drug was transported into the luminal compartment via pinocytosis, and it left the organ with the help of the luminal flow; this process was represented as the Qretetestis, Qepididymis, Qvasdeferens, Qseminalvesicle, and Qprostategland. The movement of seminal fluid/luminal fluid through the lumen of these reproductive organs served as the medium connecting each organ. This framework captured the complex dynamics of mAb distribution within the male reproductive system, providing insights into drug behavior within these tissues.

#### 2.5.1. Testis Compartment

The testis is the key male reproductive organ made up of two main components: the interstitium and the seminiferous tubules. The seminiferous tubules, which occupy around 80% of the testis, are lined by the seminiferous epithelium, which is made up of two kinds of cells—germ cells and Sertoli cells. Germ cells serve as the precursors to sperm, progressing through multiple stages of development before maturing into spermatozoa. Sertoli cells, along with the tight junction, form the blood–testis barrier (BTB), creating a selective barrier that regulates the movement of substances into the seminiferous tubules and preserves the specialized microenvironment essential for germ cell development [10]. To capture the anatomical and functional complexity of the testis, we divided the testis into 4 compartments i.e., vascular, interstitial, blood–testis barrier, and luminal. Additionally, the rete testis is included as a separate compartment, representing the point where all seminiferous tubules converge. Once the drug enters the interstitial compartment, the drug can exit the interstitial compartment via lymphatic flow, or it can be distributed into the blood–testis barrier with the help of pinocytosis. Once it reaches the Sertoli cell, it binds to the FcRn receptor and forms a complex with the FcRn, after which it is either recycled to the interstitium or into the luminal compartment. Unbound drugs within the Sertoli cells are subject to degradation; this process is governed by a degradation rate constant (Kdeg). In the luminal compartment, the drug is distributed to the rete testis via pinocytosis. From the rete testis, the drug exits the organ through luminal flow, which is represented by the flow rate (Qretetestis), and is distributed into the lumen of the epididymis, where further distribution and potential elimination processes occur.

#### 2.5.2. Epididymis Compartment

The epididymis is the second key organ in the male reproductive system, consisting of a highly convoluted structure that is anatomically divided into three regions: the Caput, Corpus, and Cauda. Each of these regions is lined with an epithelial barrier composed of various cell types, including principal cells, myoid cells, halo cells, basal cells, and apical cells. These cells, along with tight junctions, form a robust barrier that prevents the entry of toxic substances into the organ. In addition to the epithelial barrier, the epididymis contains interstitial tissue composed of connective tissue, as well as a luminal space [10]. To maintain simplicity in the model, all three regions are grouped into one region, which is further divided into four distinct compartments: vascular, interstitial, blood–epididymal barrier, and luminal. The drug that reaches the interstitium of the epididymis is taken up by the epithelial cells through pinocytosis, where it binds to FcRn receptors located in the blood epididymal barrier. Upon binding, a minor fraction of the drug is recycled back into the interstitial space, while the majority is transported across the epithelial barrier into the luminal compartment. Once in the lumen, the drug is carried away by luminal flow, represented as Qepididymis, and is eventually transported to the lumen of the vas deferens.

#### 2.5.3. Vas Deferens Compartment

The vas deferens is a tubular structure that connects the epididymis to the seminal vesicle and is composed of a smooth muscle layer and an epithelial lining that surrounds a central luminal compartment. The epithelial barrier features microvilli that extend throughout the lumen, increasing the surface area for absorption and transport [11]. To accurately model the distribution of mAbs within the vas deferens, we divided the organ into five compartments: vascular, interstitial, muscular, epithelial, and luminal.

Once the drug reaches the interstitium, it is either transported into the muscular compartment via pinocytosis or into the luminal compartment facilitated by lymphatic flow. The reflection coefficient is incorporated to account for the resistance posed by the muscular and epithelial layers. From the muscular compartment, the drug moves into the epithelial layer, where it binds to FcRn receptors, forming an FcRn–antibody complex. A portion of the drug is recycled back into the muscular compartment and then to the interstitium, while the majority crosses the epithelial barrier into the luminal compartment. Additionally, the unbound drug within the epithelium undergoes elimination through a first-order degradation process, characterized by the rate constant kdeg. From the luminal compartment, the drug exits the lumen via luminal flow Qvd and is distributed into the lumen of the seminal vesicle.

#### 2.5.4. Seminal Vesicle Compartment

The seminal vesicles are a pair of male accessory reproductive glands located from the posterior into the interior of the urinary bladder. They develop as an extension of the vas deferens at its junction with the urethra. The excretory duct of the seminal vesicles joins the ampulla of the vas deferens and merges with the verumontanum of the prostatic urethra. Structurally, the seminal vesicles consist of epithelial and muscular layers surrounding a central luminal compartment [12]. To model the distribution of mAbs within the seminal vesicles, we divided the organ into four key compartments: vascular, muscular, epithelial, and luminal. The drug distribution within the seminal vesicles follows a pathway similar to that of the vas deferens. After reaching the interstitium, the drug is transported into the epithelial layer, where it binds to FcRn receptors located in the epithelium. Following binding, the drug is either recycled back into the interstitium or transported into the luminal compartment. From the lumen, the drug is carried by the luminal flow, represented by the parameter Qseminalvesicle. Eventually, the drug is distributed into the lumen of the prostate gland.

#### 2.5.5. Prostate Gland Compartment

The prostate gland is an accessory organ located centrally between the seminal vesicles. It is organized into an irregular network of epithelial ducts embedded within connective tissue known as the stroma [13]. This ductal system consists of columnar epithelium and features a wide lumen. We divided the entire prostate gland into five compartments: vascular, interstitial, muscular, epithelial, and luminal. The drug distribution in the prostate follows the same pathway as that described for other organs. Once the drug reaches the luminal compartment, it is removed with the help of luminal flow from the prostate gland, as represented by the parameter Qprostategland.

### 2.6. PBPK Model Equations

The general equations used to describe the distribution of mAbs within the male reproductive system are outlined below. These equations are used to capture the PK of mAbs across various compartments (i.e., vascular, interstitial, epithelial, and luminal), accounting for key processes, such as pinocytosis and FcRn binding. By integrating organ-specific anatomy and physiological factors, the equations provide a comprehensive framework to simulate the movement of mAbs in male reproductive tissues, including the testis, epididymis (EP), vas deferens (VD), seminal vesicle (SV), and prostate gland (PG). A glossary of all the parameters used in the equation is described in Table S1.

**Plasma Compartment:**

$$Vplasma\frac{dCplasma}{dt}=\left(\left(PLQ_{Heart}-L_{Heart}\right)*C_{Heart}^{V}\right)+\left(\left(PLQ_{Kidney}-L_{kidney}\right)*C_{kidney}^{V}\right)+\left(\left(PLQ_{muscle}-L_{muscle}\right)*\;C_{muscle}^{V}\right)+\left(\left(PLQ_{skin}-L_{skin}\right)*C_{skin}^{V}\right)+\left(\left(PLQ_{Brain}-L_{brain}\right)*C_{brain}^{V}\right)+\left(\left(PLQ_{Adipose}-L_{Adipose}\right)*C_{Adipose}^{V}\right)+\;\left(\left(PLQ_{Thymus}-L_{Thymus}\right)*C_{Thymus}^{V}\right)+(\left(PLQ_{Liver}-L_{Liver}\right)+\left(PLQ_{spleen}-L_{spleen}\right)+\left(PLQ_{pancreas}-L_{pancreas}\right)+\;\left(\begin{array}{c} PLQ_{Sint}-L_{sint} \end{array}\right)+\left(PLQ_{Lint}-L_{Lint}\right)*C_{Liver}^{V})+\left(\left(PLQ_{Bone}-L_{Bone}\right)*C_{Bone}^{V}\right)+\left(\left(PLQ_{testis}-L_{testis}\right)*C_{testies}^{V}\right)+\;\left(\left(PLQ_{ep}-L_{ep}\right)*C_{ep}^{V}\right)+\left(\left(PLQ_{vd}-L_{vd}\right)*C_{vd}^{V}\right)+\left(\left(PLQ_{sv}-L_{sv}\right)*C_{sv}^{V}\right)+\left(\left(PLQ_{PG}-L_{PG}\right)*C_{PG}^{V}\right)+\;\left(\left(PLQ_{other}-L_{other}\right)*C_{Other}^{V}\right)+\left(L_{lymphnode}*C_{lymphnode}\right)-(PLQ_{Lung}*C_{plasma})$$

**Blood Cell Compartment:**

$$\begin{array}{cc} V_{BC\frac{dC_{BC}}{dt}} & =\left(BCQ_{HEART}*C_{HEART}^{BC}\right)+\left(BCQkidney*C_{Kidney}^{BC}\right)+\left(BCQmuscle*C_{muscle}^{BC}\right)+\left(BCQ_{skin}*C_{skin}^{BC}\right)\\ & \;\;\;\;+\left(BCQ_{brain}*C_{brain}^{BC}\right)+\left(BCQ_{Adipose}*C_{Adipose}^{BC}\right)+\left(BCQ_{Thymus}*C_{Thymus}^{BC}\right)\\ & \;\;\;\;+\left(\left(BCQ_{Liver}+BCQ_{spleen}+BCQ_{pancreas}+BCQ_{Sint}+BCQ_{Lint}\right)*C_{liver}^{BC}\right)+\left(BCQ_{Bone}*C_{bone}^{BC}\right)\\ & \;\;\;\;+\left(BCQ_{Testis}*C_{testis}^{BC}\right)+\left(BCQ_{ep}*C_{ep}^{BC}\right)+\left(BCQ_{VD}*C_{VD}^{BC}\right)+\left(BCQ_{SV}*C_{sv}^{BC}\right)+\left(BCQ_{PG}*C_{PG}^{BC}\right)\\ & \;\;\;\;-(BCQ_{lung}*C_{BC}) \end{array}$$

**Lymph Node Compartment**:

$$V_{lymphnode}\frac{dClymphnode}{dt}=(\left(1-\delta_{Heart}\right)*L_{Heart}*C_{Heart}^{IS})+\left(\left(1-\delta_{\_Heart}\right)*L_{Heart}*C_{Heart}^{IS}\right)+\left(\left(1-\delta_{Kideney}\right)*L_{kidney}*\;C_{kidney}^{IS}\right)+\left(\left(1-\delta_{\_muscle}\right)*L_{muscle}*C_{muscle}^{IS}\right)+\left(\left(1-\delta_{Thymus}\right)*L_{Thymus}*C_{Thymus}^{IS}\right)+\left(\left(1-\delta_{Liver}\right)*L_{liver}*C_{liver}^{IS}\right)+\;\left(\left(1-\delta_{spleen}\right)*L_{spleen}*C_{spleen}^{IS}\right)+\left(\left(1-\delta_{\_pancreas}\right)*L_{pancreas}*C_{pancreas}^{IS}\right)+\left(\left(1-\delta_{sint}\right)*L_{sint}*C_{sint}^{IS}\right)+\;(\left(1-{\delta \_}_{Lint}\right)*L_{Lint}*C_{L.int)}^{IS}+(\left(1-\delta_{Bone}\right)*L_{Bone}*C_{Bone}^{IS})+\left(\left(1-\delta_{Testis}\right)*L_{Testies}*C_{Testis}^{IS}\right)+(\left(1-\delta_{ep}\right)*L_{ep}*C_{ep}^{IS}+\;\left(\left(1-\delta_{VD}\right)*L_{VD}*C_{VD}^{IS}\right)+\left(\left(1-\delta_{sv}\right)*L_{sv}*C_{sv}^{IS}\right)+\left(\left(1-\delta_{pg}\right)*L_{pg}*C_{pg}^{IS}\right)+\left(\left(1-\delta_{lung}\right)*L_{lung}*C_{lung}^{IS}\right)-\;(L_{lymphnode}*C_{lymphnode})$$

**Male Reproductive System:**

**Vascular Space Plasma:**

$$V_{i}^{V}\frac{dC_{i}^{v}}{dt}=\left(PLQ*C_{J}^{V}\right)-\left(\left(PLQ_{i}-L_{i}\right)*C_{i}^{V}\right)-\left(\left(1-\delta_{i}^{V}\right)*L_{i}*C_{i}^{V}\right)-\left(CL_{upi}*C_{i}^{V}\right)+(CL_{upi}*FR*C_{i}^{Ebound})$$

**Vascular Space Blood Cell:**

$$V_{i}^{BC}\frac{dC}{dt}=BCQ_{i}*(C_{j}^{BC}-C_{i}^{BC})$$

**Endosomal Space, mAB Unbound to FcRn:**

$$\frac{dC_{i}^{Eunbound}}{dt}=(\frac{CL_{up}}{V_{E}^{i}}*\left(C_{i}^{V}+C_{i}^{IS}\right)-K_{on}^{FcRn}*C_{i}^{Eunbound}*FcRn_{i}+K_{off}^{FcRn}*C_{i}^{Ebound}-K_{deg}*C_{i}^{Eunbound})$$

**Endosomal Space, mAb Bound to FcRn:**

$$\frac{dC_{i}^{Ebound}}{dt}=K_{on}^{FcRn}*C_{i}^{Eunbound}*FcRn_{i}-K_{off}^{FcRn}*C_{i}^{Ebound}-\frac{FR*CL_{upi}}{V_{E}^{i}}*C_{i}^{Ebound}-\frac{(1-FR)*CL_{upi}}{V_{E}^{i}}*C_{i}^{Ebound}$$

**Interstitial Space:**

$$V_{int}^{i}\frac{dC_{i}^{IS}}{dt}=\left(\left(1-\delta_{i}^{V}\right)*L_{i}*C_{i}^{V}\right)-\left(\left(1-\delta_{i}^{IS}\right)*L_{i}*C_{i}^{IS}\right)-\left(CL_{\begin{array}{c} upi \end{array}}*C_{i}^{IS}\right)+\left(CL_{upi}*\left(1-FR_{ep}\right)*C_{i}^{Ep}\right)\;\;\;\;\;\;\;+\left(CL_{upi}*\left(1-FR\right)*C_{i}^{Ebound}\right)-CLupi*C_{i}^{IS}-CL_{upep\;}*C_{i}^{IS}-\left(\left(1-\delta_{i}^{ep}\right)*L_{i}*C_{i}^{ep}\right)$$

**Endosomal Space of Epithelia, mAb Unbound to FcRn:**

$$\frac{dC_{i}^{Ep_{unbound}}}{dt}=\left(\frac{CL_{up}}{V_{ep}^{i}}*C_{i}^{V}\right)-K_{on}^{FcRn}*C_{i}^{Ep\_unbound}*FcRn_{i}+K_{off}^{FcRn}*C_{i}^{Ep\_bound}-K_{deg}*C_{i}^{Ep\_unbound}$$

**Endosomal Space of Epithelia, mAb Bound to FcRn:**

$$\;\;\;\;\;\;\;\;\frac{dC_{i}^{Ep\_bound}}{dt}=K_{on}^{FcRn}*C_{i}^{Ep_{unbound}}*FcRn_{i}-K_{off}^{FcRn}*C_{i}^{Ep_{bound}}-\frac{CL_{upi}*\left(1-FR_{epi}\right)*C_{i}^{Ep_{bound}}}{V_{i}^{Ep\_bound}}\;-\frac{CL_{upi}*\left(1-FR_{epi}\right)*C_{i}^{Ep_{bound}}}{V_{i}^{Epbound}}$$

**Luminal Space:**

$$V_{i}^{LU}\frac{dC_{i}^{Lu}}{dt}=CL_{upi}*\left(1-FR_{epi}\right)*C_{i}^{Ep_{bound}}-Q_{i}*C_{i}^{Lu}+\left(\left(1-\delta_{i}^{ep}\right)*L_{i}*C_{i}^{ep}\right)$$

**Rete Testis:**

$$V_{i}^{retetestis}\frac{dC_{retetestis}}{dt}=CL_{upi}*C_{retetestes}-Q_{retetestis}*C_{retetestes}$$

**Observed Concentration of Tissue:**

**For Testis adn Epididymies:**

$$Ci^{observed}=\frac{\left(C_{i}^{V}*V_{i}^{V}\right)+\left(C_{i}^{BC}*V_{i}^{BC}\right)+\left(\left(C_{iunbound}+C_{i}^{bound}\right)*V_{i}^{E}\right)+\left(C_{i}^{IS}*V_{i}^{is}\right)+\left(\left(C_{i}^{epunbound}+C_{i}^{epbound}\right)*V_{i}^{Ep}\right)+((C_{i}^{Lu}*V_{i}^{Lu})}{V_{i}^{V}+V_{i}^{BC}+V_{i}^{E}+V_{I}^{EP}+V_{i}^{Lu}}$$

**For Seminal Vesicle, Prostate Gland, and Vas Deferens:**

$$Ci^{observed}=\frac{\left(C_{i}^{V}*V_{i}^{V}\right)+\left(C_{i}^{BC}*V_{i}^{BC}\right)+\left(\left(C_{iunbound}+C_{i}^{bound}\right)*V_{i}^{E}\right)+\left(C_{i}^{IS}*V_{i}^{is}\right)+\left(\left(C_{i}^{epunbound}+C_{i}^{epbound}\right)*V_{i}^{Ep}\right)+(C_{i}^{mu}*V_{i}^{mu})+((C_{i}^{Lu}*V_{i}^{Lu})}{V_{i}^{V}+V_{i}^{BC}+V_{i}^{E}+V_{I}^{EP}+V_{i}^{mu}+V_{i}^{Lu}}$$

### 2.7. PBPK Model Parameters

Physiological parameters for tissues, except the male reproductive systems, were obtained from Shah and Betts [9]. The lymph flow in each tissue was assumed to be 200 times lower than the plasma flow, while the endosomal volume was assumed to be approximately 0.5% of the total tissue volume. The rates of pinocytosis and exocytosis in the endosomal space of the vascular endothelium, as well as FcRn-mediated recycling and kdeg, were assumed to remain constant across all organs. For organs in the male reproductive systems that expressed FcRn in the epithelial compartment, uptake clearance (CLup) was assumed to be the same as the vascular endothelial cells, except for the testes, where the Clup values in the epithelium and the endothelium were assumed to be different. Physiological parameter values specific to the male reproductive system in a mouse, such as volumes and blood flows, were gathered through an extensive literature search [14,15,16,17,18,19], as shown in Table 1.

### 2.8. Model Fitting and Simulation

To improve the model’s ability to capture the observed data for the testes and epididymis, several assumptions were made. For the testes, it was initially assumed that the rate of pinocytosis differed between the endothelium and the blood–testis barrier (BTB). These rates were estimated by fitting the data in Monolix, and the results confirmed that the clearance (CLup) in the endothelium was distinct from that in the BTB, supporting the assumption of differential pinocytosis rates in these regions. Additionally, it was assumed that the FcRn concentration in the BTB was lower than that in endothelial cells, further refining the model. For the epididymis, the BEB volume was estimated to improve model fitting. This value was subsequently checked for physiological relevance to ensure it accurately reflected the organ’s characteristics, leading to more accurate predictions. In contrast, the parameters for vas deferens, seminal vesicles, and prostate glands were not modified.

The model was employed to estimate unknown physiological parameters such as luminal flow from each organ: Qretetestis, Qepididymis, Qvas deferens, Qseminalvesicle, and Qprostategland. The initial values for these parameters were sourced from literature or scaled from bigger animals to ensure their physiological relevance.

Parameters were estimated by fitting the mouse PBPK model to the observed PK data using the Monolix 2023R1. The following error model was used.

**Var(t) = (δintercept + δslope. Y(t))^2^**

Above, the variance Var(t) was associated with the model output Y(t) using 2 variance parameters, δintercept and δslope.

The final model structure and the parameter estimates were determined using standard model fitting criteria: visual inspection, observed v/s predicted plot, and the CV% of the estimated parameter.

The accuracy of model-simulated PK profiles was calculated by comparing model-predicted and observed AUC values and calculating the percentage predictive error (%PE) using the following equation.

$$\%PE=\left(\frac{AUC_{pred}}{AUC_{obs}}-1\right)\times 100$$

In above equation, AUC pred is the AUC obtained from model predicted PK profile and AUC obs is the AUC of the observed PK profile.

#### Translation of the PBPK Model to Bigger Animals

The whole-body PBPK model with the male reproductive systems was translated to bigger animals: rats, monkeys, and humans. In this process, key parameters that are species-specific were adjusted accordingly. Physiological volumes and blood flows for the male reproductive systems were obtained through a comprehensive literature search or calculated using allometric scaling. These parameters used for scaling up the model are shown in Table 2. Since only plasma PK data for antibodies were available in these species, and because there were no data available to describe the PK of mAbs in the reproductive system in bigger animals, the model was used to capture the plasma PK of antibodies in rats, monkeys, and humans. The model was also used to simulate the PK of antibodies in the male reproductive organs of these species. These model predictions could be validated when tissue PK data become available in the future.

### 2.9. Sensitivity Analysis

Qretetestis, Qvd, Qep, QSV, and QPG represent the flow of seminal plasma through the lumens of each respective male reproductive organ. Since precise physiological values for these parameters were not available in the literature, we used our model to estimate them. To assess the sensitivity of each parameter on mAb concentrations across various tissues and plasma in the mice model and to evaluate their impacts on overall model performance, we conducted a local sensitivity analysis. This involved varying parameter values by ±20% and calculating the AUC for both increased and decreased values. The altered AUC values were compared with the initial values to determine the percentage change. The resulting percentage change in AUC provided insight into the model’s sensitivity to fluctuations in each parameter, helping identify key drivers of system behavior.

$$\%Change=\frac{AUC_{sim}-AUC_{20}\%}{AUC_{sim}}$$

Above, AUC_sim_ represents the AUC obtained with the optimized set of the parameter and AUC_20_% represents the AUC obtained by increasing or decreasing the parameter by 20%.

## 3. Results

### 3.1. Antibody Production

The antibody for mouse biodistribution study was successfully produced and the purity of the antibody was acceptable as assessed by the SDS–PAGE (Figure S2). The resulting bands in SDS–PAGE were compared to a molecular weight ladder for analysis. Under reducing conditions, two bands were observed: one at ~25 kDa, corresponding to the antibody’s light chain, and another at ~50 kDa, corresponding to the heavy chain. Under non-reducing conditions, a single band appeared at ~150 kDa, representing the intact antibody. These results confirmed the expected molecular compositions of the antibodies under both reducing and non-reducing conditions.

### 3.2. Bioanalytical Method Development

A sandwich ELISA method was optimized to measure the concentrations of the mAbs in male organs. The assay’s lower limit of quantification (LLOQ) was approximately 1 ng/mL, while the upper limit (ULOQ) was 500 ng/mL. The coefficients of variation (CV%) values for the standards in male plasma and tissues were within ±20% of the nominal value. For all plasma and tissue samples analyzed, the CV% values were within ±30%. The concentrations of the unknowns were determined by comparing them to the standard curve.

### 3.3. PK of mAb in the Male Reproductive System

**Figure 3:**
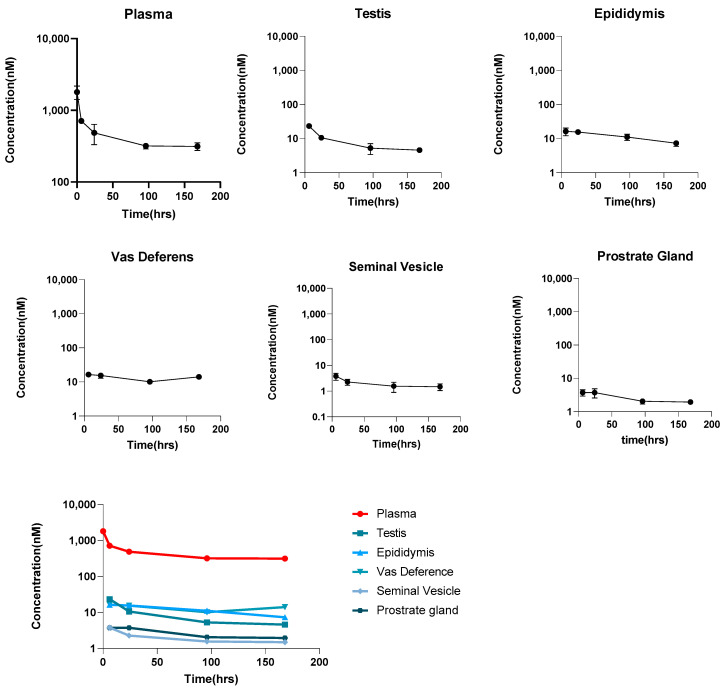
Observed concentrations of the mAbs in plasma, testes, epididymis, prostate gland, vas deferens, and seminal vesicles. Error bars represent the standard deviations in the data. The data were collected following a single 10 mg/kg intravenous dose of trastuzumab. Plasma samples were collected at 5 min, 6 h, 24 h, 96 h, and 168 h post-administration, while tissue samples were collected from the male reproductive organs after perfusion at 6 h, 24 h, 96 h, and 168 h. All samples were analyzed using an optimized sandwich ELISA.

Figure 3 presents the profiles of the observed plasma concentration versus time along with the error bar for plasma and various male reproductive organs, including the testis, epididymis, vas deferens, seminal vesicle, and prostate gland. Considering the relatively low variability observed in the data, it can be inferred that interanimal variability is minimal. This is because of the lack of antigen binding of the antibody selected here and the homogeneity of the biological system across different mice. Consequently, the average concentration data are expected to provide a reliable estimate of the antibody’s concentration within the male reproductive system. This suggests that using the mean concentration across animals will likely yield accurate predictions for the drug’s distribution in this specific tissue. To better understand the mAb distribution in each tissue, we calculated the antibody distribution coefficients. Table 3 summarizes the values of the %ABC. The data show that, although the testis exhibits a higher initial drug concentration compared to the epididymis and vas deferens, it has a lower ABC value. This discrepancy can be attributed to the lower expression of FcRn in the blood–testis barrier (BTB) [23], which leads to the reduced salvage of the antibody within the testis. In contrast, compared to other male reproductive organs, seminal vesicle and prostate gland display significantly lower drug distributions compared to plasma, with ABC values of around 0.42% and 0.52%, respectively. In general, the distributions of antibodies in the male reproductive organs are very low compared to most of the organs, as evident by the ABC values ranging from 0.5–2.6% compared to the values of 5–15% for most organs (except brain, whose ABC value is 0.35%) [8].

### 3.4. Mouse PBPK Model Fitting

Plasma and reproductive organ PK data obtained from male mice were used to fit the male PBPK models. The following parameters were estimated: epididymal fluid flow (Qep), seminal vesicle flow (Qsv), vas deferens flow (Qvd), rete testis flow (Qretetestis), and prostate gland flow (Qpg). The estimated parameter values along with their standard errors and CV% are shown in Table 4.

**Figure 4:**
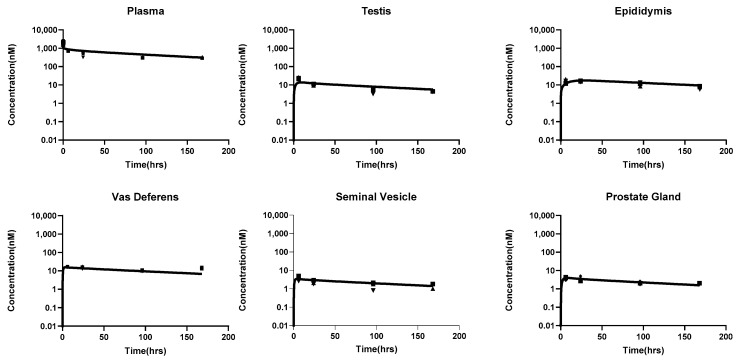
PBPK model fitted PK profile of mAbs in plasma and male reproductive organs of mice. The solid line represents the model-simulated curve, while the solid dots indicate observed data points.

As shown in Figure 4, the model was able to capture the observed data reasonably well. Simulations were performed using the estimated flow parameters, and the area under the curve (AUC) was calculated based on model-predicted concentrations. By using the model-predicted AUC and the observed AUC, the prediction error was calculated for plasma and all male reproductive organs. The prediction error analysis (Table 5) demonstrated that the model accurately captured the concentrations in plasma, testis, vas deferens, prostate gland, epididymis, and seminal vesicles, with a maximum %PE value of 24%.

#### PBPK Model Translation to Bigger Animals

**Figure 5:**
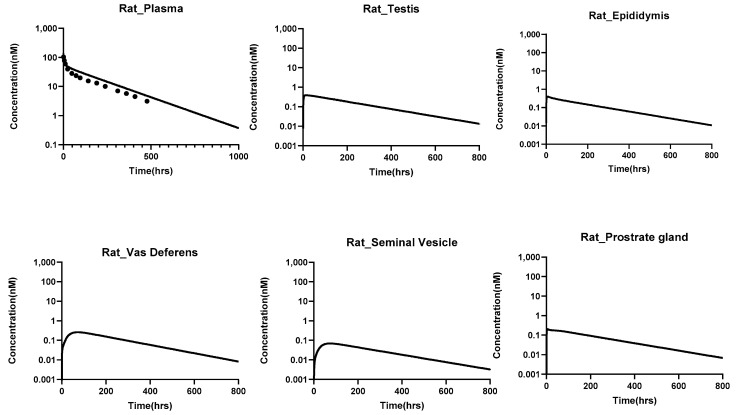
PK of antibodies in plasma and male reproductive tissues of **rats**, simulated using the translated PBPK model for antibodies that contain the male reproductive system. The simulations were performed at a dose of 0.7 mg/kg, and validation of the plasma pk was done with the help of the digitized data obtained from the literature [25].

**Figure 6:**
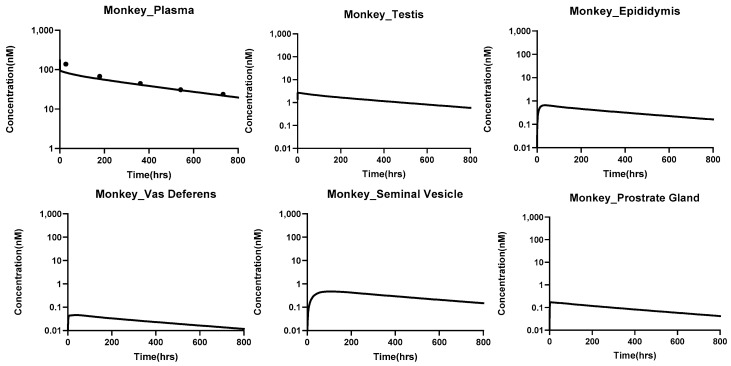
PK of antibodies in the plasma and male reproductive tissues of monkeys simulated using the translated PBPK model for antibodies that contain the male reproductive system. The simulations were performed at a dose of 1 mg/kg, and validation of the plasma pk was done with the help of the digitized data obtained from the literature [26].

**Figure 7:**
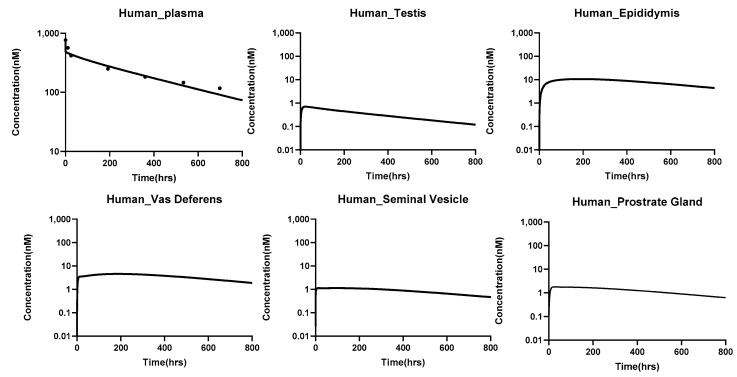
PK of antibodies in the plasma and male reproductive tissues of **humans**, simulated using the translated PBPK model for antibodies that contain the male reproductive system. The simulations were performed at a dose of 5 mg/kg, and validation of the plasma pk was done with the help of the digitized data obtained from the literature [27].

The translated PBPK model accurately captured the plasma pharmacokinetics of antibodies across species, including rats, monkeys, and humans. The percent predictive errors for plasma PK profiles calculated were 58%, 113%, and 82.8% for rats, monkeys, and humans, respectively. Figure 5, Figure 6 and Figure 7 illustrate the model-predicted antibody PK profiles for plasma and male reproductive organs in rats, monkeys, and humans, demonstrating the model’s translational capability and its potential for predicting drug distributions in human reproductive tissues.

### 3.5. Local Sensitivity Analysis

**Figure 8:**
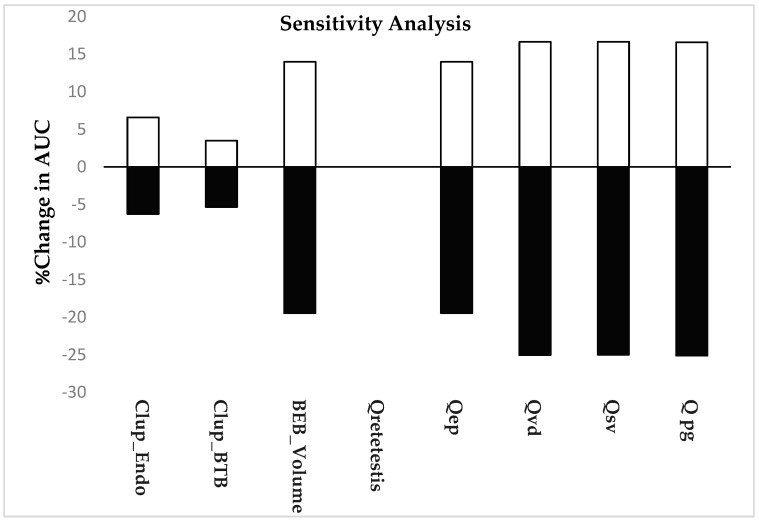
Results of a local sensitivity analysis conducted on key estimated parameters, including the CLup_BTB, BEB_Volume, Qretetestes, Qepididymis, Qvasdeferens, Qseminalvesicle, and Qprostategland of the mouse model. The goal of this analysis was to assess the impact of each parameter on the overall model performance. The sensitivity index was determined by comparing the simulated area under the curve (AUCsim), and the AUC was obtained after individually adjusting each parameter by 20%.

Figure 8 presents the results of the local sensitivity analysis, which was conducted by varying each estimated parameter by 20%. The analysis reveals that monoclonal antibody (mAb) exposure in the testis is highly sensitive to variations in the rate of pinocytosis in both the endothelium and the blood–testis barrier (BTB). In contrast, antibody exposure in the testis is least affected by changes in luminal flow from the rete testis. Among these, the rate of pinocytosis in the endothelium has a more pronounced impact on drug exposure compared to clearance uptake (CLup) in the BTB. In the epididymis, the BTB volume and luminal fluid flow are key factors influencing mAb exposure, with both causing significant changes. Conversely, in the seminal vesicle, vas deferens, and prostate, changes in luminal fluid flow significantly affect drug exposure in these organs.

## 4. Discussion

The shift towards using broad neutralizing antibodies (bnAbs) for treating HIV and Zika virus and the use of antibodies to treat reproductive system-related cancers like testicular and prostate cancer are driven by several key factors. One of the main reasons is the limitation of antiretroviral drugs, which struggle to effectively neutralize antigens due to the challenges of crossing the mucosal barrier. This barrier, found in the reproductive system, contains FcRn receptors that play a vital role in the transportation of antibodies, but conventional drugs often fail to overcome this hurdle effectively. Researchers are increasingly focusing on bnAbs because of their ability to offer broad-spectrum efficacy. These antibodies are capable of neutralizing multiple viral strains, including HIV and Zika, while also providing promising therapeutic potential for cancers of the reproductive system. Another important factor is the flexibility in antibody administration routes, which opens opportunities for localized treatment at mucosal sites, where viral transmission and cancer progression often occur. Additionally, antibodies present a novel opportunity for multipurpose prevention technologies (MPTs) [2]. These antibodies can be designed not only to prevent sexually transmitted infections but also to act as immunocontraceptives and cancer therapies, making them highly versatile.

To optimize the use of mAbs in treating various reproductive-related diseases, a clear understanding of the distribution of these antibodies across the male reproductive system is crucial. To bridge this knowledge gap, we have developed the first-ever PBPK model for antibody disposition in the male reproductive organs.

The development of the male reproductive system PBPK model followed a stepwise approach. Initially, a comprehensive literature search was conducted to gather physiological parameter values, such as organ-specific blood flow rates and tissue volumes for each component of the male reproductive system. These parameters served as the foundation for constructing a tissue-level PBPK model that was based on the true anatomy and physiology of the male reproductive organs. This tissue-specific PBPK model was then integrated into the platform whole-body PBPK model for antibodies, enabling a detailed analysis of drug distribution across the male reproductive system.

The PBPK model structures and parameters were refined using the antibody PK data in male reproductive system of mouse. A non-cross-reactive antibody trastuzumab was administered in human FcRn-expressing male mice at a 10 mg/kg dose, and plasma and male reproductive tissues were collected after perfusion. Here, the antibody used for the investigation was a typical IgG molecule that bonded to either no target or a soluble target that did not lead to any target-mediated drug distribution. It is important to perform such an investigation before understanding the PK of antibodies that bind to target to understand the effect of physiology on the distribution of antibodies in the male reproductive system. In order to make sure that the enhanced affinity of human Fc domain to mouse FcRn did not skew our analysis, all the studies were conducted in human FcRn-expressing mice. Since residual blood in tissues could contaminate the samples and lead to higher observed concentrations for antibodies, all the animals were perfused to remove the residual blood in the tissues before collecting the samples. Thus, the in vivo PK studies in mice were designed to generate high-quality data that could be used to develop the PBPK model for the male reproductive system.

Following the analysis of mouse PK data, it was found that, in general, the distributions of antibodies in male reproductive tissues were lower compared to other tissues (except the brain), with tissue exposures that were 0.5–2.6% of the plasma exposure. The epididymis and vas deferens each had a higher exposure (~2.5%) compared to the testes (~1.7%), which had a higher exposure compared to the seminal vesicle and prostate gland (~0.5%). There were physiological reasons one could derive to support such low concentrations of antibodies in the male reproductive system, which would mainly relate to vascular integrity, FcRn expression, tissue lymph flow, and epithelial barrier properties. For example, it could be hypothesized that, compared to the epididymis and vas deferens, the testis had less antibody exposure due to the moderate expression of FcRn in the BTB, which resulted in faster elimination of the drug from the testis. The observation that, in general, male reproductive tissue had low yet quantifiable exposure of antibodies following systemic administration, which could have notable impact on drug development. This observation suggested that antibody-based therapeutics (e.g., ADCs and radioimmunoconjugates) have the potential to lead to undesired pharmacology on male reproductive organs, and these tissues should be evaluated during toxicity studies. The observed PK data indicate that it is possible to develop antibody-based therapeutics for the treatment of diseases related to the male reproductive system (e.g., prostate cancer and infectious diseases) and contraceptives that alter the binding of sperm to zona pellucida. As such, the unprecedented PK data generated here can have notable implications on the discovery and development of antibody-based therapeutics.

The in-house PK data in mice were used to fit the PBPK model with the help of Monolix2023R1. To improve the model’s ability to capture the PK data of testis, few assumptions were made. We assumed that the Clup value of the BTB was different compared to that of the Clup of the endothelium. Based on the literature evidence, which states the FcRn distribution was moderate in BTB [28], we assumed that FcRn concentrations in the BTB were ten-fold lower compared to that of the endothelium (i.e., the value of the FcRn in BTB was assumed to be 4.98 × 10^−6^ M). For epididymis, we estimated the volume of the blood–epithelial barrier (BEB). The CLup_BTB, Clup_Endothelium, and BEB volume of the epididymis were first estimated with the help of the Monolix, and then later fixed to further estimate the values of the luminal flow parameters of all the organs. Model performance was evaluated by calculating the percentage prediction error (%PE), which confirmed if the model fit the experimental data well. While some instances of slight overpredictions and underpredictions were observed, no consistent trend of model deviation was detected. Additionally, predicted exposures for the observed data in each tissue remained within a two-fold range.

After successfully capturing the data from mice, the PBPK model was translated to bigger animals, including rats, monkeys, and humans. All models were validated by fitting the plasma data, and the accuracy was assessed by calculating the percentage prediction error (%PE) of the plasma curve. While the translated PBPK models in bigger animals, including humans, were able to capture the plasma PK data well, there were no tissue PK data published for antibodies in the male reproductive tissues of these species. Hence, the validation of the translated PBPK model needs to be deferred until such PK data are available.

## 5. Conclusions

We investigated the PK of the antibodies in the male reproductive tissues of human FcRn-expressing mouse. It was found that the exposure of antibodies in the testis was 1.7%, epididymis was 2.57%, in the vas deferens was 2.01%, in the seminal vesicle was 0.42%, and in the prostate gland was 0.52% of the plasma exposure. A novel translational PBPK model was developed to characterize the PK of antibodies in male reproductive systems. The model was first built using the mouse PK data, where detailed anatomical structures and physiological parameters from the literature were included, and unknown parameters were estimated using the data. The mouse model was scaled to bigger animals to develop a PBPK model that could predict the PK of antibodies in rat, monkey, and human male reproductive organs.

## Figures and Tables

**Table 1:** Physiological parameter values of the blood flows and the volumes of the mouse male reproductive organs.

ORGAN	Blood Flow (L/min)	Plasma Flow (L/min)	Vascular (L)	Endothelial (L)	Interstitial (L)	Epithelial (L)	Muscular (L)	Lumen (L)
Testis	0.00081	0.00099	7.07 × 10^−6^	1.18 × 10^−6^	1.89 × 10^−5^	1.74 × 10^−4^		3.48 × 10^−5^
Rete Testis								0.00000156
Epididymis	0.003078	0.003762	4.35 × 10^−6 (a)^	7.20 × 10^−7 (a)^	1.16 × 10^−5 (a)^	1.07 × 10^−4 (a)^		2.14 × 10^−5 (a)^
Vas Deferens	0.001179 ^(a)^	0.001441 ^(a)^	3.9 × 10^−7 (a)^	2.00 × 10^−8 (a)^	2.20 × 10^−7 (a)^	2.55 × 10^−6 (a)^	2.45 × 10^−7 (a)^	7.84 × 10^−7 (a)^
Seminal Vesicle	0.002142 ^(a)^	0.002618 ^(a)^	4.1234 × 10^−7 (a)^	1.033 × 10^−7 (a)^	0.00000144	0.000003637 ^(a)^	0.000002061 ^(a)^	0.000013 ^(a)^
Prostate Gland	0.000006921	0.000008459	4.758 × 10^−7^	1.19 × 10^−7^	0.00000547	0.00000952	6.26178 × 10^−6^	0.00000194

^(a)^ Represents the parameters calculated by the allometric scaling using the relationship Blood Flow = a(BW)^0.75^ and Volume = a(BW)^1^.

**Table 2:** Physiological parameter values of the blood flows and the volumes in the male reproductive organs of rats, monkeys, and humans [18,19,20,21,22,23,24].

ORGAN	Species	Plasma Cell Flow	Blood Cell Flow	Vascular	Endothelial	Interstitial	Epithelial	Muscular	Lumen	Rete Testis
		(L/min)	(L/min)	(L)	(L)	(L)	(L)	(L)	(L)	(L)S
**Testis**	**Rat**	4.28 × 10^−3^	3.50 × 10^−3^	4.80 × 10^−5^	8.00 × 10^−6^	2.24 × 10^−5^	1.18 × 10^−3^		1.56 × 10^−4^	8.00 × 10^−5^
	**Monkey**	1.41 × 10^−1^	1.16 × 10^−1^	2.46 × 10^−5^	1.23 × 10^−7^	5.41 × 10^−6^	1.59 × 10^−5^		1.23 × 10^−6^	1.23 × 10^−6^
	**Human**	6.90 × 10^−1 (a)^	5.64 × 10^−1 (a)^	0.00345	0.000575	0.00661	0.092		0.00661	0.00575
**Epididymis**	**Rat**	1.21 × 10^−2^	9.90 × 10^−3^	1.10 × 10^−5^	1.83 × 10^−6^	2.93 × 10^−5^	2.69 × 10^−4^		5.22 × 10^−5^	
	**Monkey**	5.34 × 10^−2^	4.37 × 10^−2^	8.55 × 10^−5^	1.43 × 10^−5^	7.92 × 10^−4^	1.14 × 10^−3^		8.17 × 10^−4^	
	**Human**	3.32 × 10^−1 (a)^	2.71 × 10^−1 (a)^	0.00537 ^(a)^	0.000895 ^(a)^	0.01432 ^(a)^	0.132 ^(a)^		0.02506 ^(a)^	
**Vas Deferens**	**Rat**	6.81 × 10^−3^	5.57 × 10^−3^	8.91 × 10^−7^	1.49 × 10^−6^	2.22 × 10^−6^	1.51 × 10^−5^	1.80 × 10^−6^	4.30 × 10^−6^	
	**Monkey**	5.34 × 10^−2 (a)^	4.37 × 10^−2 (a)^	2.67 × 10^−5^	4.44 × 10^−6^	6.67 × 10^−5^	4.95 × 10^−4^	3.36 × 10^−5^	8.67 × 10^−5^	
	**Human**	3.32 × 10^−1 (a)^	2.71 × 10^−1 (a)^	0.0001689 ^(a)^	2.815 × 10^−5 (a)^	0.001689 ^(a)^	0.0009908 ^(a)^	0.000563 ^(a)^	0.00219 ^(a)^	
**Seminal Vesicle**	**Rat**	1.31 × 10^−2^	1.08 × 10^−2^	6.87 × 10^−6^	1.15 × 10^−6^	1.60 × 10^−5^	4.03 × 10^−5^	2.29 × 10^−5^	1.42 × 10^−4^	
	**Monkey**	1.61 × 10^−1^	1.32 × 10^−1^	0.00409	2.045 × 10^−5^	8.589 × 10^−5^	0.0007198	0.000409	0.002531	
	**Human**	7.39 × 10^−1 (a)^	8.19 × 10^−1 (a)^	0.000213	0.0000353	0.000497	0.00439	0.00071	0.0012496	
**Prostate Gland**	**Rat**	2.01 × 10^−2^	1.65 × 10^−2^	6.87 × 10^−6^	1.15 × 10^−6^	1.60 × 10^−5^	4.03 × 10^−5^	2.29 × 10^−5^	1.42 × 10^−4^	
	**Monkey**	3.79 × 10^−1 (a)^	3.10 × 10^−1 (a)^	6.09 × 10^−6^	1.02 × 10^−5^	6.09 × 10^−4^	9.64 × 10^−4^	1.02 × 10^−4^	2.84 × 10^−4^	
	**Human**	1.00 ^(a)^	6.05 × 10^−1 (a)^	0.001584	0.000264	0.003696	0.03268	0.00528	0.00929	

^(a)^ Represents the parameters calculated by the allometric scaling using the relationship Blood Flow = a(BW)^0.75^ and Volume = a(BW)^1^.

**Table 3:** Calculated AUC and percentage antibody biodistribution coefficient (ABC) values for different male reproductive organs.

Organs	ABC (%)
Plasma	
Testes	1.70
Epididymis	2.57
Vas Deferens	2.01
Seminal Vesicle	0.42
Prostate Gland	0.52

**Table 4:** Parameters estimated for the mouse male reproductive organ PBPK model.

Parameter	Final Estimates	SE	CV%
Testes CLup_BTB(l/h/l)	6.78 × 10^−2^	4.30 × 10^−6^	6.34 × 10^−3^
Testies_CLup_E(l/h/l)	1.27 × 10^2^	4.10 × 10^−5^	3.23 × 10^−5^
Qretetestes(L/h)	1.20 × 10^−8^	3.40 × 10^−8^	2.83 × 10^2^
Qep(L/h)	1.60 × 10^−4^	1.10 × 10^−4^	6.88 × 10^1^
Qvd(L/h)	2.00 × 10^−5^	1.90 × 10^−6^	9.50
Qsv(L/h)	9.40 × 10^−5^	1.20 × 10^−5^	1.28 × 10^1^
Qpg(L/h)	1.60 × 10^−5^	8.60 × 10^−7^	5.38
ep_BEBV_M(L)	2.80 × 10^−4^	4.00 × 10^−5^	1.43 × 10^1^

**Table 5:** % prediction error for mouse plasma and male reproductive organs.

	Predicted (AUC_o-t)_ (h. nmol/L)	Observed (AUC_0-t)_ (h. nmol/L)	%PE
Plasma	87,505	70,799	23.6
Testis	1495	1355	10.3
Epididymies	2253	1988	13.3
Seminal Vesicle	369	355	3.77
Vas Deferens	1757	1783	1.5
Prostate gland	423	436	3.07

## Data Availability

The data presented in this study are available on request from the corresponding author.

## Supplementary Materials

The following supporting information can be downloaded at https://www.mdpi.com/article/10.3390/antib14010017/s1, Figure S1: standard curve of the ELISA. Figure S2: SDS–PAGE results. Table S1: Glossary of the parameters used in the model.
