# Supplementary material for: Investigation of Antibody Pharmacokinetics in Male Reproductive System and Its Characterization Using a Translational PBPK Model
Source: Antibodies (Basel). 2025 Feb 13;14(1):17. doi: 10.3390/antib14010017 (PMC11843977; doi:10.3390/antib14010017)
Supplement: Supplementary file 1 [file antibodies-14-00017-s001.zip › antibodies-3401853-supplementary.pdf]

Table S1. Glossary of the parameters used in the PBPK model

PARAMETERS	UNITS	DEFINITION
Q_I	L/h	Plasma Flow to the tissue i
L_i	L/h	Lymph flow to the tissue i
FcRn	M	The concentration of the FcRn in the endosomal space of the endothelium and epithelium of certain organs in the male reproductive system
K_{on}	1/hours	Association Rate constant between the FcRn and the mAb
K_{off}	1/hours	The dissociation Rate constant between the FcRn and the mAb
K_{deg}	1/hours	First Order Degradation rate constant of FcRn unbound mAb
FR		Fraction of mab that is bound to FcRn which is recycled back to the vascular, interstitium, or lumen (in male reproductive system)
CL_{UP}		Rate of pinocytosis per unit endosomal space of the epithelium and the endothelium
δ_{is}		Lymphatic Reflection coefficient
$V_{ep}^{i}, V_{int}^{i}, V_{E}^{i}, V_{i}^{LU} V_{i}^{V}, V_{i}^{LU}, V_{i}^{reteestis}, V_{i}^{mu}$	L	The volume of epithelium, interstitium, endothelium, muscular, lumen, and rete testis
δ_{i}^{V}		Vascular Reflection Coefficient
$C_{i}^{V}, C_{i}^{BC}, C_{iunbound}, C_{i}^{bound}, C_{i}^{IS}, C_{i}^{epunbound}, C_{i}^{epbound}, C_{i}^{mu}, C_{i}^{Lu}$	M	The concentration of the mab in vascular, endothelial, interstitial, epithelial, muscular and luminal compartment.
Qretetestis,<br>Qep, Qvd, QSV, QPG	L/hr	Flow of the luminal fluid from Rete-testis, epididymies, Vas Deferens, Seminal Vesicles, Prostate gland

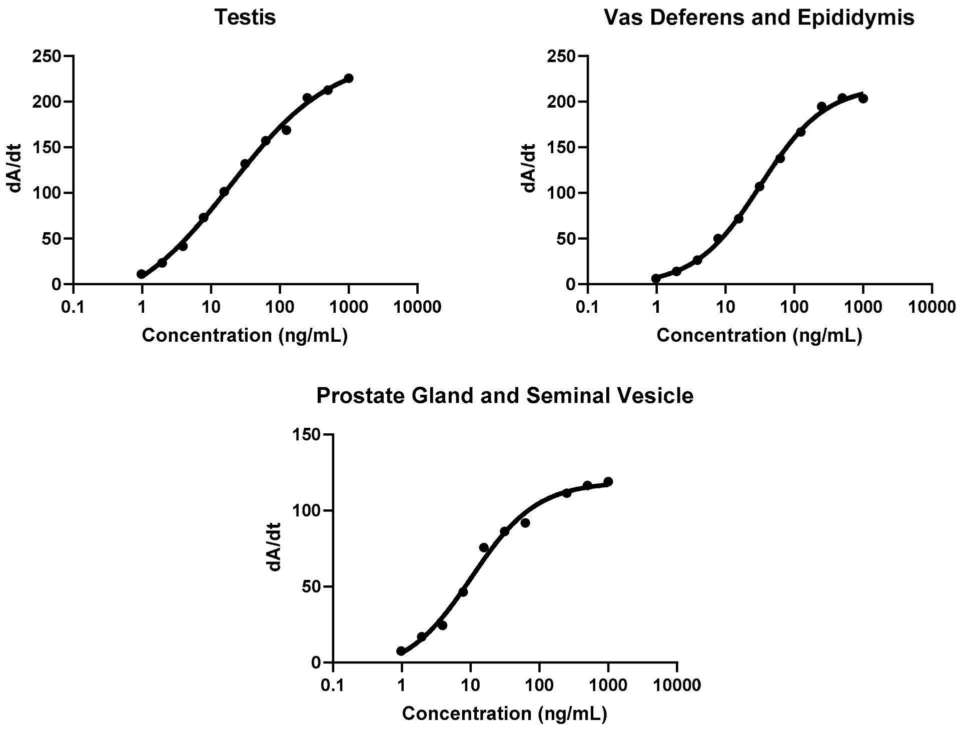

Figure S1. Standard curves for each tissue, generated using ELISA. These curves were tailored to ensure accurate quantification of the antibody concentrations specific to each tissue type. The optimization process involved adjusting dilution factors to achieve high sensitivity, precision, and reproducibility across the tissue samples.

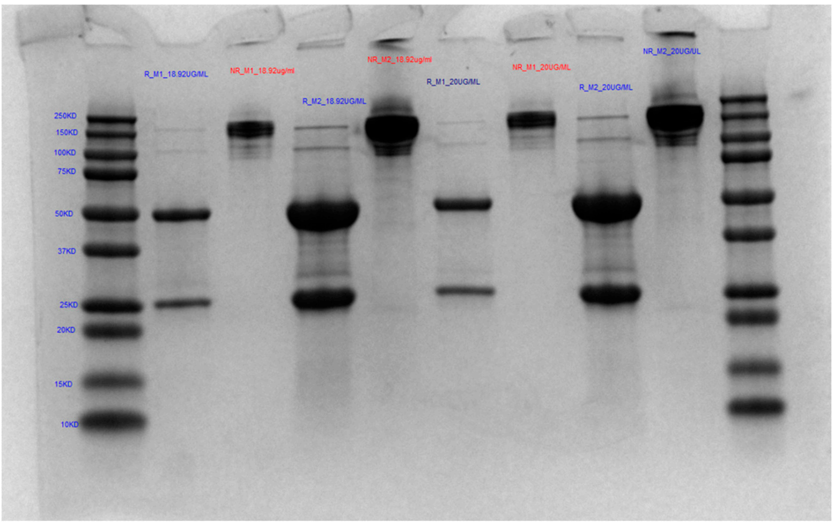

Figure S2: Figure shows the results from SDS-PAGE analysis, which reveals two distinct reducing bands—one at 50 kDa and another at 25 kDa—indicating the separation of heavy and light chains of the antibody. Additionally, a single non-reducing band is observed at 150 kDa, corresponding to the intact antibody structure under non-reducing conditions. These results confirm the antibody's integrity and expected molecular weight under both reducing and non-reducing conditions.
